# Energy Product Options for *Eucalyptus* Species Grown as Short Rotation Woody Crops

**DOI:** 10.3390/ijms9081361

**Published:** 2008-07-30

**Authors:** Donald L. Rockwood, Alan W. Rudie, Sally A. Ralph, J.Y. Zhu, Jerrold E. Winandy

**Affiliations:** 1 School of Forest Resources and Conservation, University of Florida, Gainesville, FL, USA, 32611– 0410. E-Mail: dlrock@ufl.edu; 2 USFS Forest Products Laboratory, One Gifford Pinchot Drive, Madison, WI, USA 53726–2398 E-Mails: arudie@fs.fed.us; sralph@fs.fed.us; jzhu@fs.fed.us; jwinandy@fs.fed.us

**Keywords:** *Eucalyptus*, *Eucalyptus grandis*, *Eucalyptus amplifolia*, *Corymbia torelliana*, short rotation woody crops, ethanol, biofuels, silvichemicals

## Abstract

*Eucalyptus* species are native to Australia but grown extensively worldwide as short rotation hardwoods for a variety of products and as ornamentals. We describe their general importance with specific emphasis on existing and emerging markets as energy products and the potential to maximize their productivity as short rotation woody crops. Using experience in Florida USA and similar locations, we document their current energy applications and assess their productivity as short-term and likely long-term energy and related products.

## 1. Introduction

Interest in renewable, CO_2_ neutral, and sulfur-free biomass as a clean source of fuel, chemicals and materials is accelerating [1]. Many products currently derived from petrochemicals can be produced from biomass feedstocks: lubricants, polymers, high matrix composites, textiles, biodegradable plastics, paints, adhesives, thickeners, stabilizers and a range of cellulosics [2]. Biomass can be converted into a variety of energy products and chemicals [3], including liquid fuels and electricity. Methanol, ethanol, and hydrogen may be used in advanced (fuel cell–powered) vehicles. Advanced technologies may increase alcohol conversion by fermenting cellulose. Biomass pyrolysis produces charcoal, bio-oil, and gases in varying proportions, depending on the technology and raw material. Charcoal is used for the production of pig iron and high quality steel and for the production of iron alloys, particularly in Brazil and Australia. Charcoal is also widely used as activated carbon, with some 130,000 tons of wood annually transformed into activated carbon worldwide. Phenolic derivatives from bio-oil can partly replace petrochemical phenol in phenolformaldehyde resins. Fractionating woody biomass for a wide range of products and materials has been demonstrated by a closed loop pilot plant in New Zealand [4]. After washing and pre-heating biomass, hemicellulose was hydrolyzed and the lignin and cellulose dried to produce “value-added” chemicals, hardboards, activated carbon, animal feed, or bioenergy feedstock.

Biomass and biofuels have been identified by the US Department of Energy as critical technologies for minimizing the costs of reducing carbon emissions [4]. Cofiring in coal-fired power plants, integrated gasification combined-cycle units for the forest industry, and ethanol from the hydrolysis of lignocellulosics have the most potential, with estimated annual carbon offsets in the US alone ranging between 16–24 Mt, 4–8 Mt, and 12.6–16.8 Mt, respectively, by the year 2010. The near term energy savings from the implementation of each of these technologies should cover the associated costs with cofiring giving the highest return and lowest technical risk.

Potential biomass species include some of over 700 *Eucalyptus* species, commonly known as eucalypts, that are native to Australia and its northerly islands. Eucalypts have been successful as exotics because of their capacity for fast growth and tolerance of harsh environments involving many effective adaptations: indeterminate growth, coppicing, lignotubers, drought, fire, insect resistance, and tolerance of soil acidity and low fertility. Many eucalypts have wood properties, such as high density, suitable for fuel and charcoal production, pulp and paper manufacturing, and sawn wood.

*Eucalyptus* is the most valuable and widely planted hardwood in the world (18 million ha in 90 countries [5]. Eucalypts are grown extensively as exotic plantation species in tropical and subtropical regions throughout Africa, South America, Asia, and Australia, and, in more temperate regions of Europe, South America, North America, and Australia. In 2000, India had 8.0 million mostly low productivity ha followed by Brazil with 3.0 million mostly intensively cultivated ha reaching average productivities of 45–60 m^3^/ha/year.

Of the 12.751 million ha of eucalypt plantations reported to FAO in 2005, the almost 12 million ha classified as productive forest [6] was nearly all accounted for by 12 countries (Table 1). Eucalypt planting has intensified in recent years and continues to do so, especially in tropical countries. In these regions of faster growth, rotations were as short as 5 years with yields as high as 70 m^3^/ha/yr. Eucalypts are also commercially planted in the Congo, Indonesia, Malaysia, Thailand, France, Portugal, New Zealand, and the US. China has perhaps the largest current commitment to establishing eucalypt plantations at a rate of 3,500–43,000 ha/year. Plantation area in southern China increased more than threefold from 325,000 to 1.1 million ha in 20 years.

A few eucalypt species and hybrids constitute the majority of these plantations. Most domesticated eucalypts are from the subgenus *Symphyomyrtu*s, the largest of the 10 subgenera currently recognized within *Eucalyptus*, containing over 75% of the species. Four species and their hybrids from this subgenus, *Eucalyptus grandis* (*EG*), *E. urophylla* (*EU*)*, E. camaldulensis*, and *E. globulus*, account for about 80% of the eucalypt plantations worldwide. *EG* is the most widely used species in plantation forestry worldwide in tropical and subtropical areas not only as a pure species, but also as a parental species in hybrid breeding. It has the fastest growth and widest adaptability of all *Eucalyptus* species. The greatest area of plantations of *EG* and its hybrids with other species is in Brazil and several other Central and South American countries. It has been planted extensively in India, South Africa, Zambia, Zimbabwe, Tanzania, Uganda, and Sri Lanka and is grown in California, Florida, and Hawaii in the United States. *EU* has been widely planted in the tropics for many years. *E. globulus* is the premier species for temperate zone plantations in Portugal, Spain, Chile, and Australia. For pulp production and increasingly for solid wood, *EG*, *EU*, and the *EGxEU* hybrid are the most favored in tropical and subtropical regions.

Numerous *Eucalyptus* species have been evaluated in various edaphic and climatic sites in Florida. From 1965 to 1984, US Forest Service research focused on the best of 156 seed sources of 67 species for southern Florida, resulting in >1,500 selected *EG*. Since 1979, the University of Florida evaluated nine species [7]. Current tested *Eucalyptus* genetic resources have been largely defined by major freezes from 1982 to 1989, e.g., as low as –6°C for more than 18 hours in southern Florida in January 1985. For areas of frequent freezes, testing has produced desirable genotypes of *E. amplifolia* (*EA*).

Vegetative propagation of superior clones is one key to enhancing *Eucalyptus* productivity [8–15]. Rooted cuttings commenced in the 1960s and provide adaptation to local sites with cost and wood property advantages. In Brazil, for example, 3.5 million ha of plantations support many domestic and industrial activities. In the pulpwood sector, forest productivity has increased from 12 to 40 m^3^/ha/year through breeding and silvicultural practices. Optimized and efficient transformation and recovery procedures exist for some *Eucalyptus* genotypes. Transgenic plantlets of various *Eucalyptus* species and hybrids have been regenerated from stem or leaf segments. Micropropagation and transformation have combined with efforts to engineer novel traits (exogenous genes) or to alter existing traits (modified endogenous genes or transferred homologous genes from related species). Elite hybrid clones with superior wood quality, rapid growth, and disease resistance are used extensively in tropical and subtropical regions of Brazil, South Africa, Congo, and China.

Short Rotation Woody Crop (SRWC) systems may be implemented in Florida and elsewhere. On suitable sites and/or with intensive culture, fast growing hardwoods such as *EG* and *EA* may reach harvestable size in as few as three years [16, 17]. *EG* is the most productive, largely because of the genetic improvement conducted since the late 1960s [8]. *EG* is now grown commercially in southern Florida for mulchwood [18] and can be used in central Florida, while *EA* is suitable from central Florida into the lower Southeast. *Corymbia torelliana* (*CT*) has been used as a windbreak for vegetable crops in southern Florida and is now being utilized widely for citrus windbreaks.

Eucalypts are utilized worldwide for a wide array of products including pulp for high quality paper [14], lumber, plywood, veneer, solid and engineered flooring, fiberboard [19, 20], wood cement composites [21, 22], mine props, poles, firewood, charcoal, essential oils [23–26], honey, tannin, and landscape mulch [18] as well as for shade, windbreaks, and phytoremediation [17, 27, 28]. The expansion of eucalypt plantations throughout the world is largely attributable to eucalypts’ superior fiber and pulping properties and the increased global demand for short-fiber pulp.

Elite hybrid clones are extensively used by the cellulose and paper industry because of wood quality and rapid growth. In many developing countries, eucalypt wood is important for fuel and building material in rural communities. *Eucalyptus* wood is a precursor for the preparation of activated carbon adsorbents for use in liquid-phase applications, such as water and wastewater treatment [29]. In the Congo, *Eucalyptus* is primarily grown for firewood and pulp, but is also used for round poles or sawn timber. In China, eucalypt plantations are harvested for pulpwood, fiberboard, sawlogs, roundwood, veneer, fuelwood and oil. As new high-yielding plantations mature, some 50–60% may be used for pulp and paper production. The rest will be used for plywood, medium density fiberboard (MDF) and sawn timber. Residues and leaf litter are used for fuelwood. Eucalypt oil production is primarily confined to cooler, temperate regions, where up to 150,000 ha of plantations are used for oil.

*Eucalyptus* SRWCs have shown suitability for some traditional products and may be suitable for many biofuels in Florida. Of the energy in dry whole-tree chips of 9-year-old *EG* in southwest Florida, 70% was recovered as char and oil, which could be transported and stored, and 21% was converted to noncondensed volatile oil and low-energy gas that could only be used on site or sold to an adjacent user [30]. *EA* and *EG* SRWCs are promising for cofiring in coal-based power plants in central Florida [31], but little is known about their suitability for a wider range of value-added products. Opportunities for developing technology to produce renewable bioenergy (biofuels and biopower) have recently gained momentum in the State’s public policy, research, and media.

Accordingly, the general objective of this review and the Florida-based research reported here was to determine forest biorefinery\bioenergy product opportunities for these SRWCs. The specific objectives were to evaluate and compare the broad suitability of eucalypts and specifically *EG*, *EA*, and *CT*, and estimate their suitability for a range of products.

## 2. Experimental Section

*EA*, *EG*, and *CT* from Florida were assessed for their suitability to make bioenergy products. The genotypes that were evaluated included three very good *EG* (2805, 2814, and 2817) clones and four well-tested *EA* progenies based on statewide genetic tests (Table 2). The 26.9cm DBH *EG* 2805 tree in a 11.8-year-old clonal test near Haines City, and the 19.7cm 6.7-year-old coppiced 2814 and 29.5cm 13.3-year-old 2817 in a study at Tampa were represented by 2.4m long basal logs. Four 8.3-year-old *EA* trees averaging 19.2cm in DBH in a study near Old Town were compared by basal logs; five 2.4m logs in the tree of 4836 estimated within tree variability. Four approximately 15-year-old *CT* trees in a windbreak near Clewiston with average DBH of 23.6cm were also represented by basal logs. The logs were harvested and shipped to the USFS Forest Products Laboratory (FPL) in Madison, WI, on June 27, 2007, where they were cut into 1.3m lengths to remove a midlog disk, debarked and sectioned to chippable size if needed, and put in storage at 4°C on July 2.

### 2.1. Wood and Fiber Properties

Various properties of the disks and process batches were then determined. The disks, or sections of large disks, were saturated in water, weighed green, and dried at 38°C to determine wood moisture content (MC) and specific gravity (SG) using a water displacement method. Each of the resulting 13 batches (Table 2) was chipped, usually the day before refining. Thermomechanical pulps (TMP) were prepared in a Sprout-Bauer Model 12–1CP (Andritz, Inc., Muncy, PA) 305-mm single-disk refiner with the chips held at 58 kPa for 10 minutes prior to refining. The feed rate was set to 1kg/min, and the plate gap was 0.15 mm (Sprout-Bauer refiner plates D2B503). Specific energy consumption averaged 200–250 Wh/kg. Fiber characteristics such as percent fines, pH, and fiber length were next assessed.

### 2.2. Silvichemicals

Condensates from steam pretreatment of wood chips of the 13 batches were collected and analyzed by ^13^C NMR. Approximately 50 ml of each condensate was air dried to constant weight. Dried solids (∼70 mg) were dissolved in 400 μl of DMSO-d_6_. The central solvent peak served as the internal reference, DMSO at δ_H_ 2.50, δ_C_ 39.5 ppm. Samples were run with standard Bruker pulse sequences on a Bruker DPX–250 (62.9 MHz ^13^C) spectrometer fitted with a quadranuclear 5-mm Z-gradient coil probe. Qualitative ^13^C spectral experiments were acquired with a standard power-gated sequence with a 1.0 s delay.

### 2.3. Biofuels

Chemical composition analysis of batches EG1 and EA4 (Table 2) were conducted by FPL’s Analytical and Microscopy Laboratory using an improved high performance anion exchange chromatography with pulsed amperometric detection (HPAEC-PAD) method [18]. The hydrolytic reaction was followed by measuring the carbohydrates in the hydrolyzates. For fast analysis, only glucose in the hydrolyzates was measured using a commercial glucose analyzer (YSI 2700S, YSI Inc., Yellow Springs, OH).

To evaluate the potential for bioconversion, Batch EG1 was pretreated using four processes: standard sulfuric acid method at 180°C with acid charge on oven dry wood of 1.84%, hot water at 180°C, and two FPL proprietary processes (patent pending). The pretreatment was conducted in 1-liter sealed stainless steel cylinders (pulping digesters) in an autoclave-type arrangement using steam heat. About 130 gram (od) wood chips were used under a liquid to wood ratio of 5. The duration time of pretreatment was fixed at 30 min. At the end of the pretreatment, the solid was collected and directly transferred to size reduction using a laboratory 8-inch disk refiner. Solid loss was determined from the measured wet weight and moisture content of the collected solid. Enzymatic hydrolysis was carried out at 2% of substrate (w/v) in 50-mL sodium acetate buffer using a shaker/incubator (Thermo Fisher Scientific, Model 4450, Waltham, MA) at 200 rpm. The pH and temperature were adjusted to 4.8 and 50°C, respectively. A mixture of Celluclast 1.5 L with an activity loading of approximately 15 FPU/g substrate and Novozyme 188 with an activity loading of approximately 22.5 CBU/g was made.

## 3. Results and Discussion

### 3.1. Wood and Fiber Properties

For ethanol and methanol production regardless of technology used, certain wood properties are almost universally favored: higher wood density, lower moisture content, and higher extractives content [33]. Among and within Florida-grown *EG*, *EA*, and *CT*, differences were noted for some of these properties (Table 3). *EG* was densest and *EA* lightest based on the limited genotypes and ages represented. Considerable within species variation for wood properties was evident in each species, suggesting that deployment of favorable clones would be advantageous in producing energy products. Similar variation in the characteristics of refined fibers also emphasized the importance of genetic variation in making other products.

Wood quality of the *EG* x *EU* hybrid can be evaluated at 5 years [10]. Basic density and age were positively correlated. Fiber length and wall thickness tended to increase with age. Width, lumen diameter, and chemical composition did not vary with age. Screened yield was directly related to age up to 5 and 6 years; for kappa number, an inverse relation with age was observed with a trend until 4–5 years. Different *Eucalyptus* woods had constant polysaccharide and lignin contents but differing extractive levels [36].

Small variations of pulp yield and basic density can be predicted from some chemical parameters [37]. Woods with higher pulp yield had very distinctive syringyl/guaiacyl (S/G), syringaldehyde/vanillin (S/V), and total phenols, independent of basic density. Woods with higher basic density and lower pulp yield were identical for S/V, S/G, methoxyl group, and total sugars. Methoxyl group and total sugars content were the best parameters for woods with lower density.

Wood quality traits are primary targets for genetic modification. Because lignin removal is costly and energy-consuming, reducing lignin content and modifying its composition for increased pulping efficiency are important objectives of tree engineering as an alternative to adjusting bleaching and delignification processing technologies [38]. Substantial progress has been made, aided by a growing understanding of lignin biosynthesis and its genetic control.

### 3.2. Silvichemicals

While *Eucalyptus* species contain many silvichemicals, their cost effective capture is critical to commercial use. Capturing them as an incidental byproduct of other wood processing would be advantageous.

**Figure 1.**
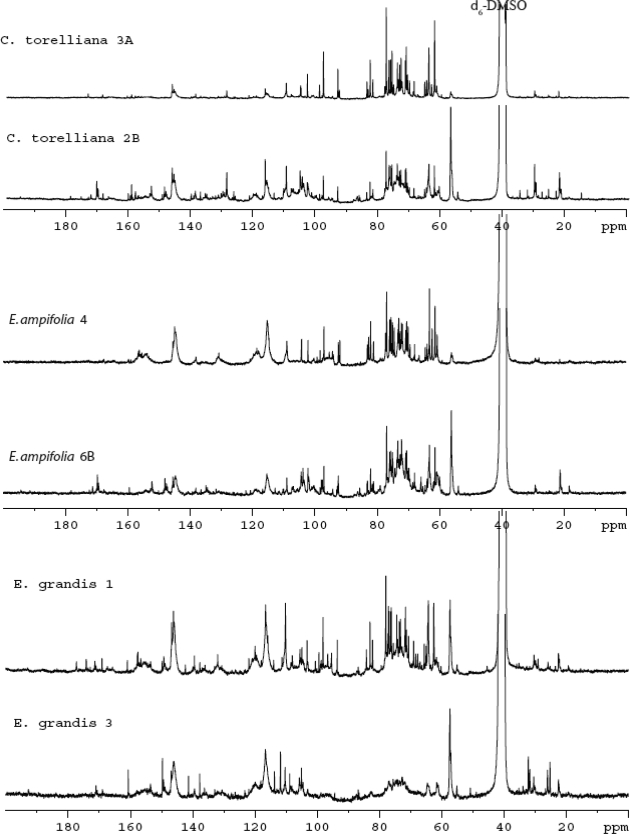
^3^C NMR spectra for condensates from two CT samples (top), two E. amplifolia samples (middle), and two EG samples (bottom).

Condensate extracts from steam pretreatment of wood chips of *EA, EG,* and *CT* contained a multitude of components with no one compound in sufficient quantity to make separation and recovery a viable commercial option. The samples ranged from 1.59 to 0.16 grams of dry material per 50 milliliters of condensate, with most between 0.3 and 1 gram. The chemical makeup differed among species, trees within species, and within trees. Figure 1 illustrates the compositional range found in select condensates from *EA*, *EG* and *CT*. From the literature [39, 40], we would expect to see chemical shift signals due to lignin/lignan moieties, and the signal at ∼ 56 ppm is generally indicative of the aromatic methoxyl for these types of compounds. The *EG* condensates all showed similar methoxyl content; however, the *EA* and *CT* condensate examples show the extremes for methoxyl content.

*Eucalyptus* in general are also known to contain significant amounts of polyphenols of the condensed (proanthocyanidins) and hydrolysable tannin (ellagitannin and gallotannin) varieties. A comparison of the chemical shifts of authentic catechin, the base structure for condensed proanthocyanidins, shows consistent data for two of the *CT* condensates. This catechin type structure was not significant in the extracts of *EA* or *EG*.

Spectra for ellagic acid (ellagitannin aglycone) compared most favorably with the *EG* condensates but was less obvious in the *EA* and *CT* extracts. The most predominant and common features for all of the condensates may be attributed to catechol and gallol type structures. Most notably the signals clustered around 145 ppm for the carbons bearing adjacent hydroxyl groups, the 118–114 ppm grouping for the protanated carbons of the catechol group, and more sharply around 110 ppm for protonated gallol carbons.

It is possible that the sharp signals indicative of carbohydrate units between 100 and 60 ppm are part of the hydrolysable tannin components and/or they may be small, water-soluble oligomers not associated with phenolic units. All of the condensates showed substantial amounts of carbohydrate signals with the exception of *EG* 3 (Figure 1). Fatty acids of potential value for biodiesel should show a pattern of aliphatic signals in the 40 to 20 ppm region which is not apparent in the condensates.

*Eucalyptus* compounds often have several roles [23], including defense against insect and vertebrate herbivores and protection against UV radiation and cold stress. Best-known are the terpenoids, which give *Eucalyptus* foliage its characteristic smell. *Eucalyptus* is also a rich source of phenolics such as tannins. While some phenolics have been the basis of past industries, the most recent interest is newly identified formylated phloroglucinol compounds (FPCs), which include euglobal, macrocarpal, and sideroxylonal subtypes. All FPCs have the same fully substituted, formylated, aromatic moiety, but vary in side chain structure. FPCs have a wide range of biological actions and play a major ecological role as powerful antifeedants.

FPCs appear to be concentrated in subgenus *Symphyomyrtus*, are absent from subgenera *Monocalyptus* and *Idiogenes* (*E. cloeziana*), and occur sparingly and at low concentrations in *Corymbia* and *Blakella* [41]. In subgenus *Eudesmia*, *E. phoenicia* appeared rich in euglobals. Of 39 *Eucalyptus* species and four *Melaleuca* species that are or could be planted in low-rainfall areas, rich sources of FPCs are some Western Australian oil mallees, most notably *E. loxophleba*, with sideroxylonal concentrations as high as 9% of the dry leaf mass. Sideroxylonal was most abundant, with high concentrations also observed in *E. cinerea*, *E. pulverulenta* and *E. mannifera*. Large quantities of macrocarpals occurred in *E. kartzoffiana* and *E. pulverulenta* as well as in some *E. viminalis*. In species with sideroxylonals, concentrations of 1,8-cineole and FPCs are strongly associated, suggesting that selection for cineole will result also in selection for FPCs. A strong correlation between foliar concentrations of 1,8-cineole and sideroxylonal in *E. polyanthemos* is important ecologically because marsupial folivores use cineole concentration as a cue to the concentration of the FPCs; if they detect high concentrations of cineole, they will eat little, if any, of the foliage. Likewise, low cineole concentrations suggest that the foliage is palatable. If confirmed in other *Eucalyptus* species, selection increasing the concentrations of essential oils might also yield similar increases in the FPCs. In contrast, eucalypts selected for rapid growth rate may contain lower concentrations of FPCs.

Conversion of 1, 8-cineole by a strain of *Aspergillus niger* produced two novel alcohols, 3-*exo*- and 3-*endo*-hydroxycineole [24]. Furthermore, hydrogenolysis of 3-*endo*-hydroxycineole afforded p-methane-3, 8-e″-diol which has been isolated as a plant growth inhibitor from *E. citriodora*.

With integrated tree crop systems in Western Australia, large volumes of high-cineole eucalyptus oil could be produced from mallee eucalypts well below current market prices [25]. Large industrial solvent markets are currently in transition following withdrawal of 1,1,1-trichloroethane due to international measures to control ozone depletion. Although penetration of these markets would need prices about half those in traditional eucalyptus oil markets, this may be achievable with economies of scale, genetic advances, and improved harvesting and processing technologies.

### 3.3. Biofuels

**Figure 2.**
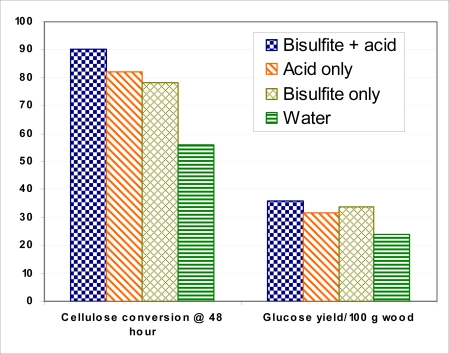
Cellulose conversion efficiency by four methods for EG1.

Two *Eucalyptus* species grown in Florida are suitable for biofuel and bioenergy production. As is the case with all hardwoods, the major chemicals in EG1 and EA4 are lignin, glucan, and xylan (Table 5). Glucan contents are about 40%, lower than other hardwood species. However, mannan contents are very low. Mannose from mannan is difficult to ferment. The main hemicellulose is xylan of 11%. Xylan can be easily removed in pretreatment and fermented. Lignin content at about 34% is higher than most hardwood species, which suggests that SRWC *Eucalyptus* species can be ideal for bioenergy production through thermoconversion. Our experiments with EG1 indicate that *Eucalyptus* species can be readily converted biologically for biofuel and byproduct production. Under mild pretreatment conditions, such as sulfuric acid pretreatment, over 80% cellulose conversion can be achieved, and over 30g glucose can be obtained from 100 grams od wood when using the two FPL processes (Figure 2).

Hemicellulose can be reduced to sugar by acid or enzymatic hydrolysis and then fermented to produce ethanol [2]. Solid energy crops such as SRWC willow, poplar, and eucalyptus can be utilized whole to produce heat and electricity directly through combustion or indirectly through conversion for use as biofuels like methanol and ethanol. The synthesis of lower viscosity methylcellulose from juvenile eucalyptus is feasible and suitable [42]. Pulping severity is an important factor for the preparation of methylcellulose. Autohydrolysis to fractionate biomass into soluble sugar oligomers and a solid residue mainly made up of cellulose and lignin followed by posthydrolysis of autohydrolysis liquors allows the generation of xylose solutions with low inhibitor content [43]. The concentrations of furfural and other sugars obtained as reaction byproducts (glucose and arabinose) were below the threshold leading to problems in further bioconversion of the liquors. Considerable ethanol production alternatives are under investigation [44–48], e.g., ethanol may be generated from spent sulfite liquor.

The use of organic liquids from carbonization of wood to produce biopitches, which can then be mixed with charcoal to make electrodes, may replace both pitch and coke from fossil sources [1]. Biomass-derived carbons may have important and successful applications. Silicon for semiconductors, new batteries, aircraft structures and composite-like metal intercalated with graphite are some specific applications where a high degree of purity is required and metal or sulfur impurities from oil and coal can contaminate the final product. Some Brazilian companies have already begun biooil recovery at a competitive price. Mild hydropyrolysis in deeper beds may be more likely to produce lighter, less oxygenated and more stable tars/oils than liquids produced at atmospheric pressure [49]. Direct liquefaction of mallee biomass is lower cost and higher efficiency transportation of the liquid biofuel to a central user or processing facility [50]. Complex xylo-oligosaccharides may be obtained by hydrothermally treated *Eucalyptus* wood [51]. Polycyclic aromatic hydrocarbons may be obtained by partitioning the liquid products of the slow pyrolysis of *E. grandis* wood [52].

Gasification of biomass residues and spent pulping liquors into syngas is expected to produce new value streams [54, 55]. Syngas could be converted into biofuels, power, chemicals and other high value materials. Growth in biomass markets worldwide is anticipated to double between 2004 and 2013. The greatest percentage growth of biomass thermal plants is expected in Asia and Latin America. Biofuels (both ethanol and biodiesel) are rapidly increasing in the US, EU, and Brazil [56]. Key drivers for worldwide biomass expansion are: 1) meeting increasing energy demands where indigenous fossil fuel sources are non-existent or in decline, 2) meeting greenhouse gas emission targets, 3) supporting domestic and industrial waste management projects, 4) utilizing forest, crop and livestock residues, 5) rising fossil fuel prices.

Methanol can be produced from biomass by gasification, gas upgrading, and eventual synthesis over a copper-zinc oxide catalyst [57]. Woody species including *Eucalyptus* tend to be less favorable for methane production because of higher cell wall constituents (86.1%) and lignin (22.6%) and lower nitrogen (0.43%) and phosphorous (0.05%) [58, 59]. Methanol, like ethanol, can be used to store chemical energy for use in fuel cells, avoiding the storage and transportation problems inherent in the direct use of hydrogen in such systems. Methanol could be an attractive transportation fuel, provided effective methanol-powered fuel cells and fuel-cell/electric vehicles are developed.

Using catalytic upgrading of pyrolytic biomass oils, yields of up to 75% oil have been obtained by low-pressure liquefaction of a variety of woody and nonwoody materials, although the resultant oils contain about 20% water and are poor fuels. Catalytic upgrading can generate a useful product [ e.g., 60], but little work has been done on scale-up.

Given the prominence of current and future *Eucalyptus* plantations (Table 1) in areas with large energy needs and biomass energy crop production potential (Table 6), *Eucalyptus* can be a significant contributor of a range of energy products. In tropical and subtropical areas where *Eucalyptus* species are already widely planted, their deployment for energy products is likely, especially when grown on non-agricultural land and without environmental impact. In Florida, *EG, EA,* and *CT* utilization may expand from the current mulchwood market to pulps and biofuels.

For example, the “Integrated Oil Mallee” project in Western Australia involves more than heat and power generation [2]. Biomass will come from growing SRWC eucalyptus mallee in 4–5m wide strips to help solve the dryland salinity problem on crop lands. Harvesting trees on a 3–4 year cycle will provide pharmaceutical oils, activated carbon, heat and power, tradeable renewable energy certificates, and even carbon credits. Larger scale plants may follow since the salinity problem extends over millions of hectares of arable land. Planting eucalypts for leaf oil may simultaneously provide: 1) a commercial incentive for vegetative restoration, 2) sustainable control of groundwater and salinity, 3) an environmentally benign substitute for a widely used solvent damaging to the ozone layer, 4) specialty chemical products, 5) biomass fuels, and 6) carbon sequestration.

Biomass-derived electricity and liquid fuels may be able to compete with fossil fuels [61–63]. The most likely short-term technology to convert biomass to electricity is integrated gasifier/gas turbine cycles, which may be more efficient than conventional coal electric power generation and coal gasification and have lower capital costs. Nevertheless, the techniques and technologies for growing biomass and converting it into modern energy carriers must be more fully developed. Suitable policy options include a reallocation of agricultural subsidies and a review of energy subsidies to create a more level playing field, internalizing external negative costs of fossil fuels by methods such as carbon taxes, and using these taxes to support bioenergy and other alternative carbon mitigation options.

Overall, bioenergy will likely be the highest contributor to global renewable energy in the short to medium term with dedicated energy crops, such as SRWC *Eucalyptus*, providing a larger proportion of the biomass feedstock in the coming decades [2]. Opportunities for energy crops include development of biorefineries, carbon sequestration, and the growing trend towards small, distributed energy systems.

## 4. Conclusions

*Eucalyptus* is widely planted and produces abundant biomass. Many conversion technologies are well understood, and several are being developed. Biomass characteristics, difficulty in securing adequate and cost effective supplies early in project development, and planning constraints currently prevent *Eucalyptus* bioenergy from reaching its full potential. However, increased biomass productivity and quality, prospects for carbon trading, distributed energy systems and hydrogen, multiple products from biorefining, and government incentives should foster *Eucalyptus* biomass use for bioenergy.

## Figures and Tables

**Table 1.** Area of productive eucalypt plantations and semi-natural forests (*) in 2005 by country, species, and age class [6].

		Area (1,000 ha) by Age Class (years)					
Country	Species	0–5	5–10	10–20	20–30	30–40	>40
RSA	nitens	109.7	99.3	19.4	0.7	1.8	
	grandis	144.1	140.7	44.9	3.7	1.7	
Sudan	spp	118.2	189.1	165.5	8.0		
China	spp	683.0	576.4	982.7	154.4		
India	spp	43.0	64.4	103.2			
	spp*	656.1	984.2	1,576.0			
Myanmar	camaldulensis	1.1	2.1	2.2	1.1	0.5	
Vietnam	spp	222.4	286.5	67.1	7.0	3.0	
Iran	spp	24.6	6.2				
Italy	spp	7.0	8.2	8.2			
Australia	regnans	5.2	0.2	2.8	3.7	4.7	1.1
	globulus	131.2	260.1	48.7	1.1	0.4	
	pilularis	5.2	5.5	0.5	1.4	4.6	0.4
	dunnii	5.3	12.2	0.2			
	grandis	5.2	5.5	0.5	1.4	4.6	0.4
Argentina	grandis	15.8	32.6	34.5	11.8	3.9	
Brazil	spp	2118.1	756.5	121.0	30.3		
Chile	spp	353.4	204.1	85.4	7.2	2.0	
	Subtotal	4,648.6	3,633.8	3,262.8	231.8	27.2	1.9
	Total			11,806.1			

**Table 2.** *EG*, *EA*, and *CT* genotypes (their age in years, number of trees, and number of logs per genotype) and resulting numbers of stem disks and batch numbers in the FPL study.

Species	Genotype (Age, No of trees, No of Logs)	Disks	Batches
*EG*	2805 (11.8,1,1), 2814(6.7,1,1), 2817(13.3,1,1)	3	EG1, EG2, EG3
*EA*	4836 (8.3,1,5), 4543 (8.3,1,1), 4853 (8.3,1,1), 4875 (8.3,1,1)	6	EA1, EA2, EA3, EA4, EA5, EA6
*CT*	? (∼15,1,1), ? (∼15,1,1), ? (∼15,1,1), ? (∼15,1,1)	4	CT1, CT2, CT3, CT4

**Table 3.** Variation in log specific gravity (kg/m^3^) and moisture content (%) and batch fines (%), pH, and fiber length (mm) from Florida-grown *EG*, *EA*, and *CT* basal logs.

Species	Genotype (Batch)	Age (yrs)	No of Trees	Specific Gravity	Moisture Content	Fines	pH	Fiber Length
***EG***	**3 clones**	**10.6**	**3**	**544**	**107**	**38.9**	**4.05**	
	2805 (EG1)	11.8	1	522	104	30.3	3.96	-
	2814 (EG2)	6.7	1	470	129	32.1	4.30	.673
	2817 (EG3)	13.3	1	640	89	54.1	3.92	-
***EA***	**4 progenies**	**8.3**	**4**	**508**	**108**	**59.5**	**3.97**	
	4836 (EA5)	8.3	1	527	107	53.1	3.89	-
	4843 (EA6)	8.3	1	469	115	53.5	3.89	-
	4853 (EA1)	8.3	1	506	109	70.7	-	-
	4875 (EA4)	8.3	1	529	88	60.5	4.11	.502
***CT***	**4 trees**	**15**	**4**	**526**	**101**	**50.0**	**4.20**	
	? (CT1)	15	1	526	80	48.6	4.17	-
	? (CT2)	15	1	610	98	52.6	4.20	-
	? (CT3)	15	1	555	94	37.1	4.23	.472
	? (CT4)	15	1	411	131	61.5	4.21	-

**Table 5.** Carbohydrate characterization for two Florida eucalypts.

Species Batch	AI Ash	K. Lignin	ASL	Arabinan	Galactan	Rhamnan	Glucan	Xylan	Mannan
EG1	−0.001	0.324	0.034	0.003	0.009	0.002	0.397	0.114	0.003
EA4	−0.002	0.345	0.034	0.004	0.013	0.002	0.374	0.111	0.005

**Table 6.** Projected energy crop areas in 2025 by region and globally [2].

Region	Energy crop area (ha × 10^3^) under four IPCC scenarios			
	B1	A1b	B2	A2
North America	14992	31004	41132	34985
Central America	3406	6489	10047	7550
South America	8521	8722	15687	8219
Northern Africa	182	0	0	0
Western Africa	182	257	142	102
Eastern Africa	101	137	80	53
Southern Africa	549	791	1376	706
OECD Europe	7266	19681	17886	15092
Eastern Europe	514	1826	2715	1647
Former USSR	3534	8916	7296	6092
Middle East	526	0	0	0
South Asia	5788	12469	12726	5171
East Asia	10068	18097	21609	12163
Southest Asia	1854	4501	7406	3521
Oceania	198	537	1594	1057
Japan	650	1150	1510	855
World	58332	114577	141206	97212
